# The First Anti-Snakebite and Hepatoprotective Characterization of a Trypsin Kunitz-like Inhibitor (EcTI) from the Plant *Enterolobium contortisiliquum;* A Case of Two Soul Mates Meeting

**DOI:** 10.3390/ph16040632

**Published:** 2023-04-21

**Authors:** Caroline R. C. Costa, Mariana N. Belchor, Airam Roggero, Laila L. Moraes, Ricardo Samelo, Isabelly Annunciato, Camila R. Bonturi, Maria L. V. Oliva, Sergio F. Sousa, Marcos A. de Oliveira, Marcos H. Toyama

**Affiliations:** 1 Center of Natural and Human Sciences, Federal University of ABC (UFABC), Santo André 09210-580, SP, Brazil; 2 Biosciences Institute of Paulista Coast Campus (IB/CLP), University of São Paulo State (UNESP), São Vicente 11330-900, SP, Brazil; 3 National Institute of Pharmacology (INFAR), Federal University of São Paulo (UNIFESP), São Paulo 04044-020, SP, Brazil; 4 Unit of Applied Biomolecular Sciences (UCIBIO), REQUIMTE-BioSIM-Medicine Faculty, Porto University, 4050-345 Porto, Portugal

**Keywords:** serine protease, Cdtsp-2, Kunitz-type inhibitor, *Crotalus durissus terrificus*

## Abstract

Snake venom serine protease (SVSP) interferes with the regulation and control of important biological reactions in homeostasis and can be classified as an activator of the fibrinolytic system and platelet aggregation. Our group has recently isolated a new serine protease from *Crotalus durissus terrificus* total venom (Cdtsp-2). This protein exhibits edematogenic capacity and myotoxic activity. A Kunitz-like EcTI inhibitor protein with a molecular mass of 20 kDa was isolated from *Enterolobium contortisiliquum* and showed high trypsin inhibition. Thus, the objective of this work is to verify the possible inhibition of the pharmacological activities of Cdtsp-2 by the Kutinz-type inhibitor EcTI. To isolate Cdtsp-2 from total *C. d. terrificus* venom, we used three-step chromatographic HPLC. Using the mice paw edema model, we observed an edematogenic effect, myotoxicity and hepatotoxicity caused by Cdtsp-2. In vitro and in vivo experiments showed that the alterations in hemostasis caused by Cdtsp-2 are crucial for the development of marked hepatotoxicity and that EcTI significantly inhibits the enzymatic and pharmacological activities of Cdtsp-2. Kunitz-like inhibitor may be a viable alternative for the development of ancillary treatments against the biological activities of venoms.

## 1. Introduction

**Figure 1:**
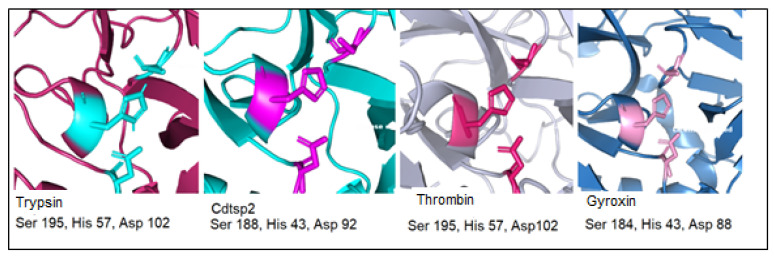
The active sites of the serine proteases are highly conserved regardless of their origin.

Snake venoms exhibit several toxic enzymes, including serine proteases, which catalysis the cleavage of peptide covalent bonds in proteins. These macromolecules play key roles in several biological processes ranging from digestion to control and regulation of blood coagulation, immune system modulation, besides exhibiting an essential control of inflammation. Serine proteases from snake venoms are trypsin-like enzymes with highly conserved S1 substituents, as we can see in Figure 1. They show high selectivity towards molecular substrates, such as blood coagulation factors, while also being glycosylated molecules; this allows them to interact with other molecules [1,2]. Several experimental data show that these proteins exhibit various pharmacological roles, including a clear pro-inflammatory action [2,3,4]. Recent studies with Gyroxin, a venom serine protease, have shown that this enzyme can act on PAR (protease activated receptor) receptors, which activate the phospholipids breakdown in the membrane and generate IP3 upon proteolytic cleavage [5,6,7].

Costa and collaborators isolated Cdtsp-2 (*Crotalus durissus terrificus* serine protease 2) with a 98% purity and characterized this protein as another serine protease different from Gyroxin. This new macromolecule causes edema by enzymatic cleavage of PAR1 receptors, PAR2 and G proteins, activating PLC and PKC, which raise cPLA2 activity by increasing arachidonic acid metabolism and its interaction with oxidative stress. Thus, the evidence suggests that this toxin is an essential and still neglected aggravating factor for the action of snake venom and the venom of other animals. Reviews on clinical manifestations induced by the bite of *Crotalus durissus* venom show that besides the neurotoxic, myotoxic and hemorrhagic action, there are also reports on the hepatotoxic potential of rattlesnake venom. Several data from experimental animal studies indicate that components of the coagulation cascade, particularly coagulation factor Xa and thrombin, lead to profibrogenic events, leading to liver fibrosis and acute liver inflammation [1,2,3,7,8,9,10,11,12]. 

Antivenoms cannot neutralize the pharmacological actions of serine proteases, therefore the search for inhibitors is fundamental to allow for adequate treatment following ophidian accidents [13,14]. Serine protease inhibitors (SPIs) are widely distributed in living organisms, such as bacteria, fungi, plants and humans, and their main activity is to regulate proteolytic activity. In plants, SPIs are involved in the control of endogenous proteolytic processes, such as in the regulation of proteases in seeds. Batista et al. [15] isolated a Kunitz-type inhibitor named EcTI (*Enterolobium Contortisiliquum* trypsin inhibitor), with 97% purity, from seeds, using a plant extract with saline and acetone solution, with four chromatographic steps. This protein exhibited 173 amino acids, four of which were cysteine, forming two disulfide bridges. As could be observed in this study, EcTI was not efficient at inhibiting all the serine proteases to which it was exposed; it managed to block the activity of trypsin, but did not have the same success with thrombin. The literature shows that EcTI has anti-inflammatory activity and acts in modulating cytokines, inducing apoptosis of breast cancer cells [16]. In addition, numerous studies reveal that EcTI exhibits anti-inflammatory activity, including in lung tissues, modulates the metabolism of various tumor cells, including in brain tumors, considerably reducing their activities [2,12,13,14,15].

Hence, this work aimed to characterize the Cdtsp-2 hepatic inflammatory effect performing the first set of experimental and in silico data of the EcTI inhibitor action on the enzymatic, edematogenic, myonecrotic and hepatotoxic effects induced by serine protease Cstsp2 from *Crotalus durissus terrificus* venom. 

## 2. Results

**Figure 2:**
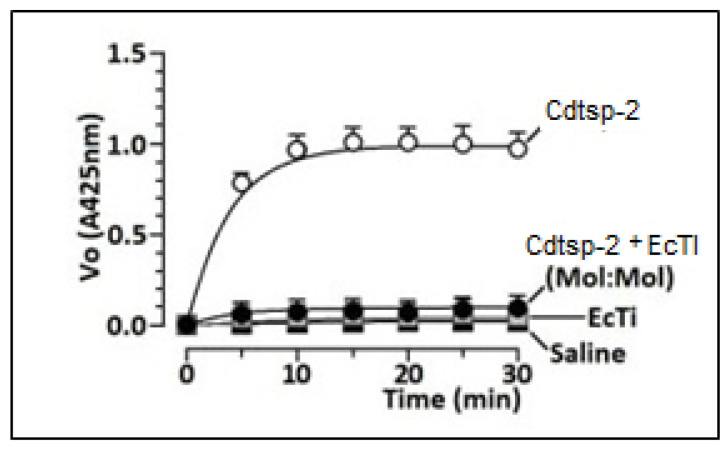
Graph of the enzymatic assay of Cdtsp-2 incubated with pure compounds and with EcTI protein (velocity × time). Prasa et al.’s [18] protocol was used, where the wavelength for detection was 405 nm and the protein substrate BapNa. The treatments were controlled, with substrate only, Cdtsp-2 incubated with substrate, Cdtsp-2 with protein, which were pipetted in microplate and incubated at 37 °C. The readings were performed on SPECTRA MAX (Molecular Devices, San Jose, CA, USA). It is observed that a decrease in the enzymatic activity of the protein was evident in the presence of EcTI.

**Figure 3:**
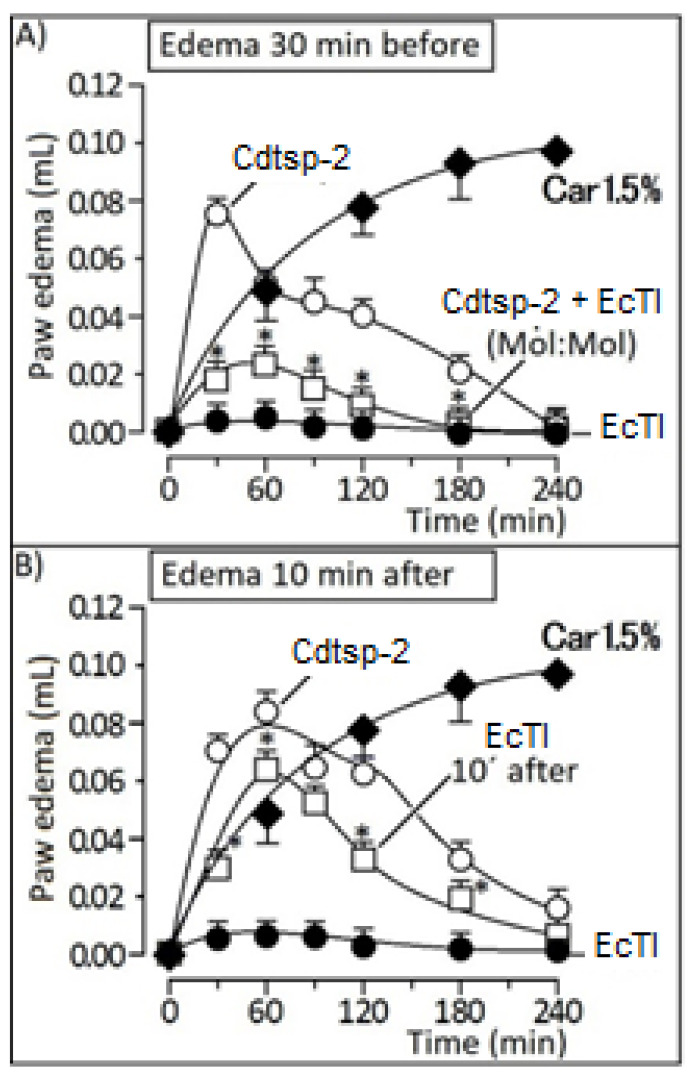
(**A**): Graph of paw edema volume (µL) of Swiss mice during inflammation, induced by the presence of Cdtsp-2 venom or the toxin with EcTi, which was injected 30 min before protein application. (**B**): Plot of the paw edema volume (µL) of Swiss mice during inflammation, induced by the presence of Cdtsp-2 or the toxin with EcTi injected 10 min after the protein application. Both assays were performed using 10 µg of protein and 50 µg of EcTi. Results were expressed as mean ± standard deviation (*n* = 5), and treatments with significant difference marked with (*) (Two-way ANOVA, with Bonferroni as a posteriori test, F = 150.0 and *p* < 0.001).

**Figure 4:**
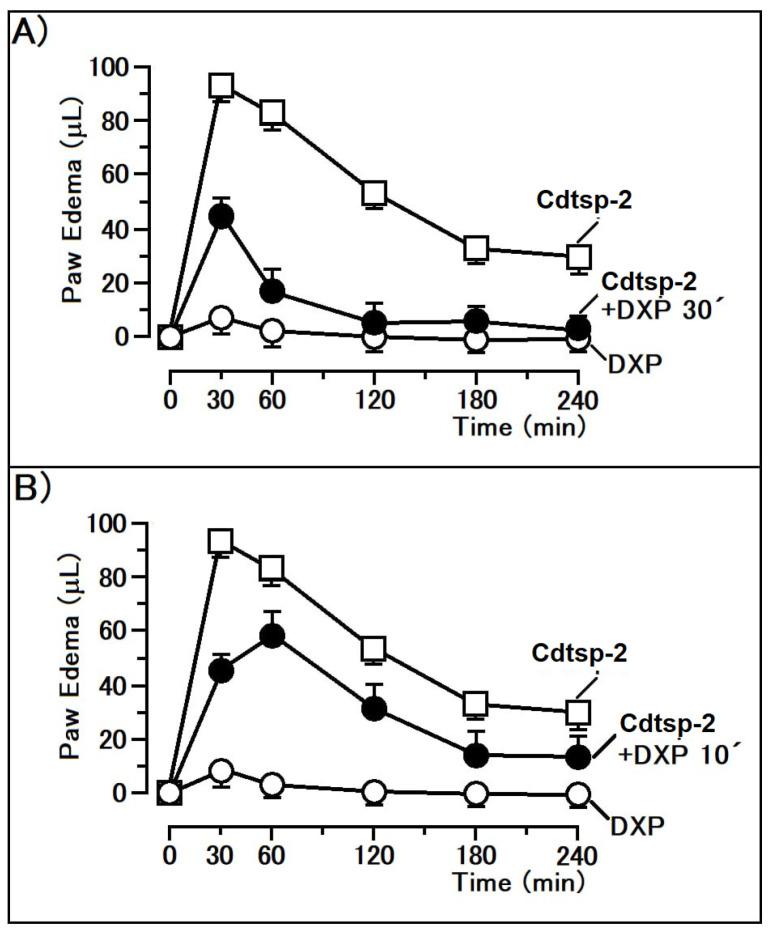
(**A**): Graph of paw edema volume (µL) of Swiss mice during inflammation, induced by the presence of Cdtsp-2 venom or the toxin with DXP (Dexametasone), which was injected 30 min before protein application. (**B**): Plot of the paw edema volume (µL) of Swiss mice during inflammation, induced by the presence of Cdtsp-2 or the toxin with DXP injected 10 min after the protein application. Both assays were performed using 10 µg of protein and 50 µg of DXP. Results were expressed as mean ± standard deviation (*n* = 5), and treatments with significant difference marked with (*) (Two-way ANOVA, with Bonferroni as a posteriori test, F = 150.0 and *p* < 0.001).

Firstly, enzymatic activity of Cdtsp-2 incubated with the Kunitz-like inhibitor EcTI, was evaluated. The result shown in Figure 2 demonstrate that EcTI abolished the enzymatic activity of the serine protease Cdtsp-2 (positive control). The second step was an evaluation of the anti-inflammatory capacity of EcTI through the edematogenic (paw edema) assay against Cdtsp-2 (positive control). In this test we used pure EcTI and Cdtsp-2 and all samples had their concentrations adjusted to 1 mg/mL. The EcTI effect was performed in two situations: applied 10 min before Cdtsp-2 injection and 30 min before Cdtsp-2 application. In the first edematogenic assay, 10 min after injecting Cdtsp-2 in the right paw, 50 µL EcTI was injected into the peritoneum of the mice, simulating a parenteral treatment. Under these conditions, we can observe in Figure 3 that EcTI inhibited the inflammatory process in a subtle way (Figure 3B). However, to verify the protective capacity against the inflammation caused by Cdtsp-2, we tested again the paw edema model, in which the inhibitor was injected into the peritoneum 30 min before Cdtsp-2 application in the paw. For control, saline solution (0.9% NaCl) was used in both analyses and all the concentrations were adjusted to 1 mg/mL. In Figure 3A, we can clearly see that EcTI has a significant anti-inflammatory protective capacity. In Figure 4, we did the same test as in Figure 3, but we used the anti-inflammatory Dexamethasone (DXP), a Phospholipase A 2 (PLA) inhibitor, and we can see that DXP was a protective molecule for paw edema as EcTI.

**Figure 5:**
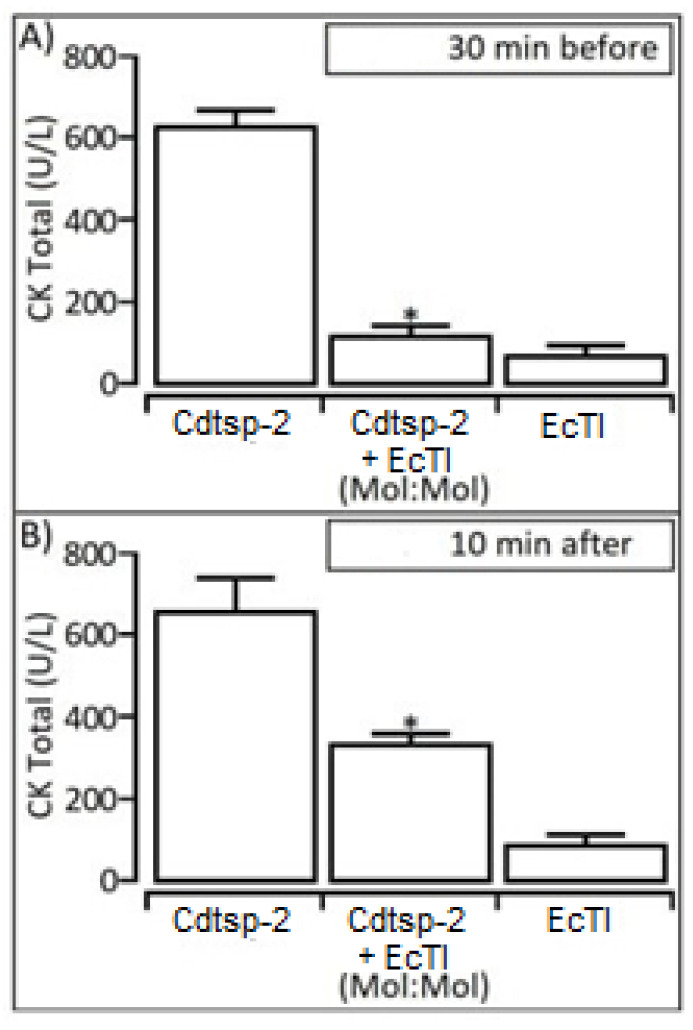
(**A**): Creatine kinase breakdown assay, revealing a significant decrease in the myotoxity of the protein when it was applied EcTI 30 min after Cdtsp-2. (**B**): There was no significant decrease in the myotoxicity of the protein when it was applied 10 min after the pure compounds. However, there was a decrease in myotoxicity when Cdtsp-2 was exposed to EcTI. Data were expressed as mean and standard deviation, and treatments with significant difference marked with (*) and ANOVA was used as the statistical test, with Dunnet as the a posteriori test, F = 9.797, *p* = 0.0017.

To analyse the myotoxic and protective capacities of the Kunitz-like inhibitor EcTI and Cdtsp-2, we performed the Creatine Kinase (CK) breakdown assay, with the concentrations adjusted to 1 mg/1 mL. Myonecrosis induced by Cdtsp-2 is shown both in the test 10 min after (Figure 5B) and in the test 30 min before (Figure 5A). The results with the Kunitz-type inhibitor showed a myoprotective capacity in both tests, significantly minimizing CK levels in both situations, which used the inhibitor 30 min before Cdtsp-2 application (Figure 5A) or applied it 10 min after the serine protease (Figure 5A). Due to these results we chose to investigate the hepatotoxicity of Cdtsp-2 and the EcTI hepatoprotective capacity against serine protease. 

**Figure 6:**
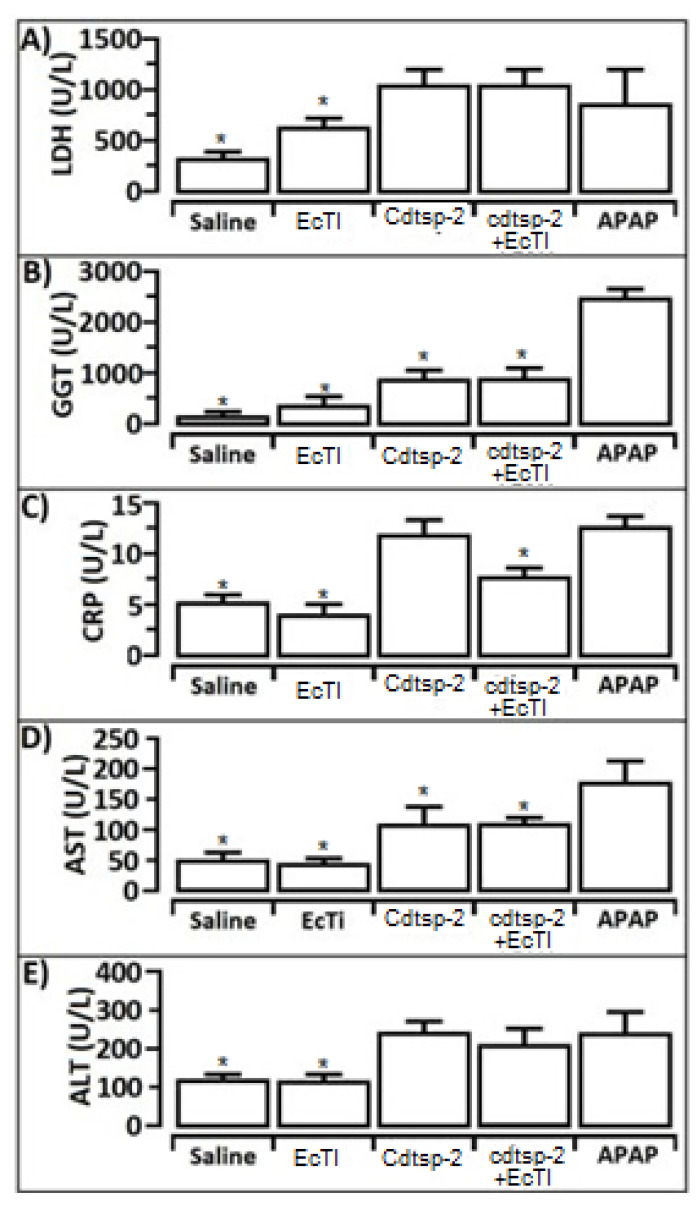
(**A**): It is possible to see an increase in LHD excretion in the presence of Cdtsp-2 protein when it was applied to EcTI 30 min after Cdtsp-2. (**B**): There was a significant decrease in Hepatotoxicity in relation to y-GT excretion caused by the protein when it was applied to EcTI 30 min after Cdtsp-2. (**C**): There was a significant decrease in liver inflammation caused by Cdtsp-2 protein when the same was applied to EcTI 10 min after Cdtsp-2. (**D**,**E**): There was no significant decrease in hepatotoxicity of the protein when it was applied to EcTI 10 min after Cdtsp-2. Data were expressed as mean and standard deviations, and treatments with significant difference marked with (*) and ANOVA was used as a statistical test, with Dunnet as a posteriori test, F = 9.797, *p* = 0.0017.

**Figure 7:**
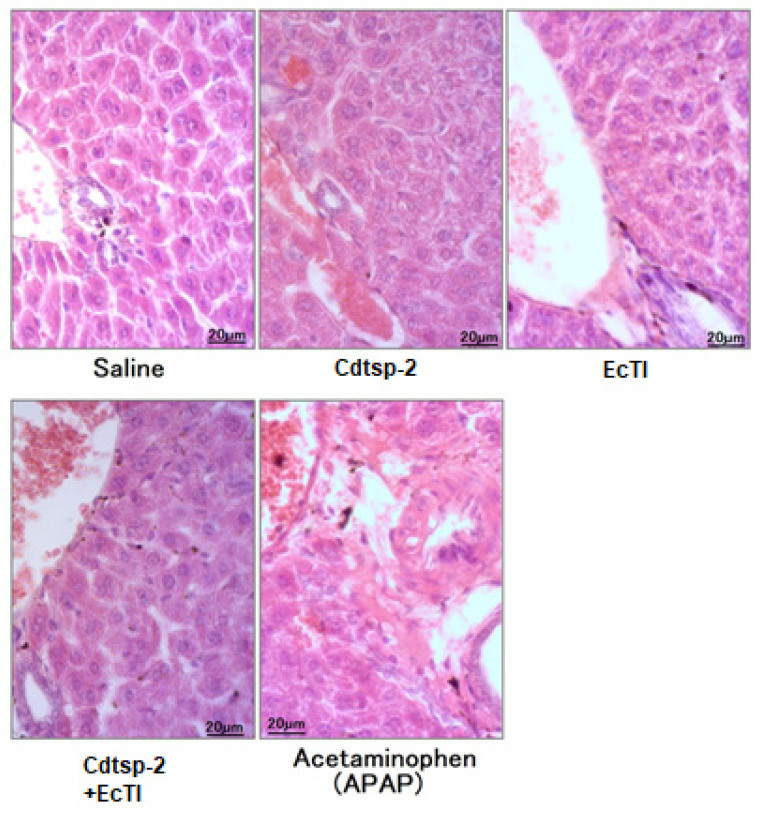
All samples present normal aspects, with neither infiltrated inflammatory or signals of necrosis. The pieces were processed according to the method described by Medeiros et al. for tests [19] and histopathological evaluations of the liver were made qualitatively with the aid of a light microscope in a blinded trial, based on the guidelines issued by the Society for Toxicologic Pathology [20] and the National Toxicology Program Health and Human Services [21].

Acetaminophen (paracetamol, N-acetyl-p-aminophenol) was used as a positive control for hepatotoxicity in all tests. In Figure 6A, the first hepatotoxic marker tested was LDH (Lactate dehydrogenase), and in this test we can see that pure EcTI is not hepatotoxic when compared to paracetamol; however, Cdtsp-2 shows LDH levels above paracetamol. Another result we can see in Figure 6A is that EcTI was not able to mitigate LDH release against serine protease. Still in reference to the hepatotoxicity, we used five different hepatotoxicity marker tests, of which the second was the Gamma-Gt presented in Figure 6B. In the graph, we observe that the Kunitz-type inhibitor was not able to mitigate the release of the y-GT molecule against Cdtsp-2. Figure 6C exhibits the release of C-reactive protein (CRP), revealing that the inhibitor was able to reduce the damage caused by serine protease. AST and ALT were also evaluated, and Figure 6 shows that, in both tests, EcTI revealed no hepatotoxic activity itself, but could not significantly reduce hepatotoxicity caused by Cdtsp-2. However, neither infiltrated inflammatory or signals of necrosis can be seen with the samples present normal aspect, as shown in Figure 7.

**Figure 8:**
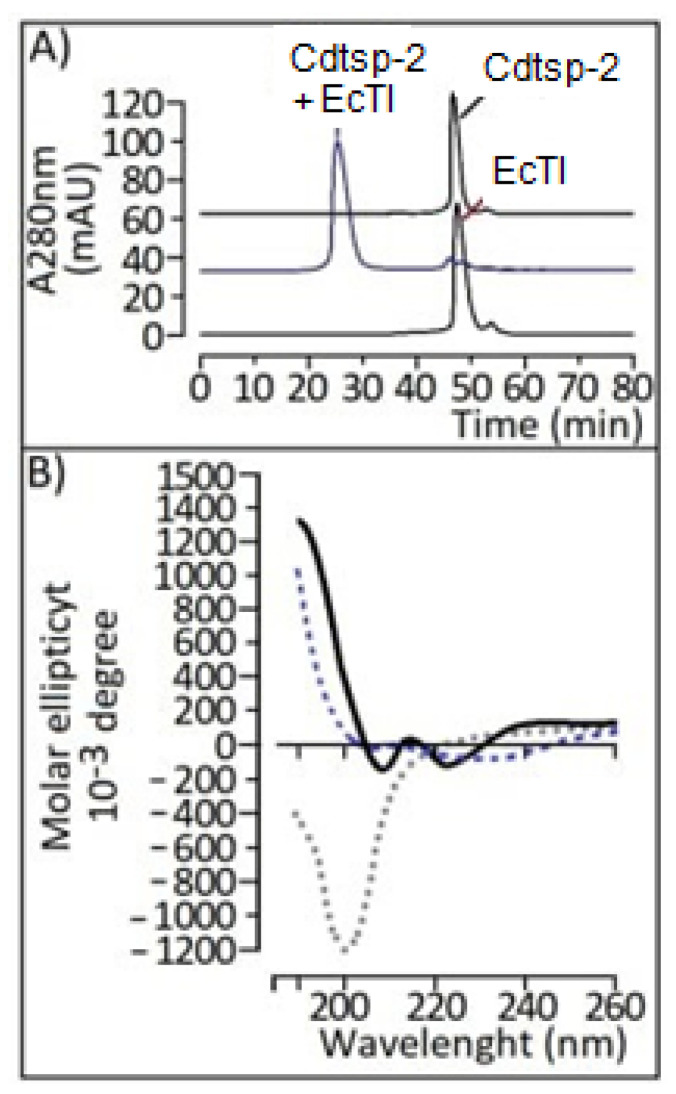
(**A**): Analysis of interaction through HPLC molecular exclusion. (**B**): Analysis on bench circular dichroism, Jasco, with monitoring at 190 to 260 nm in 0.1%TFA and 66% ACN buffer and the protein with concentration 0.02 mg/mL. Profile analysis (recording the differentiated absorption of polarized light) was given by Spectra manager program: “Randon coil” about 61% of the structure, 17.84% of the structure is organized in alpha-helices; 21.16% in sheet.

**Figure 9:**
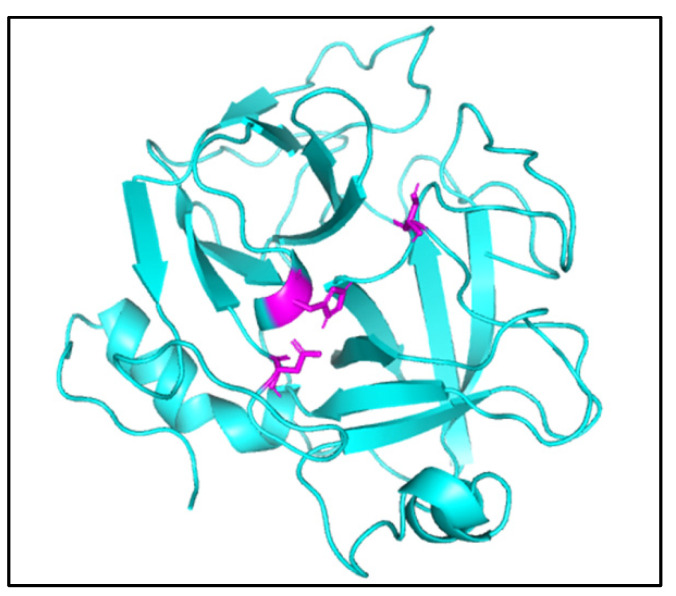
The theoretical model of Cdtsp-2 generated by PyMol. The amino acid residues referring to the active site are represented in purple.

The circular dichroism data exhibited in Figure 8B reveals that EcTI interacts so strongly with Cdtsp-2 that it significantly modifies its three-dimensional structure, changing the percentages of the secondary structures of the serine protease. A theoretical model of the *Crotalus durissus terrifucus* serine protease (Cdtsp-2) (Figure 9) was generated through homology of the *Agkistrodonhalys* thrombin-similar protein (identity 72.77%) coordinates of the enzyme (pdbcoordinates 4e7n) [12]. The structures represented were generated using PyMOL. It is possible to verify the estimated structures by dichroism, whose random coil is the most expressive. The structure was validated using MolProbity software, generating the Ramachandra graph. The quality validation of the theoretical model by Ramachandra resulted in the presentation of only three amino acids in unfavorable positions, thus the theoretical model generated by our group is viable for studies in bioinformatics interactions.

**Figure 10:**
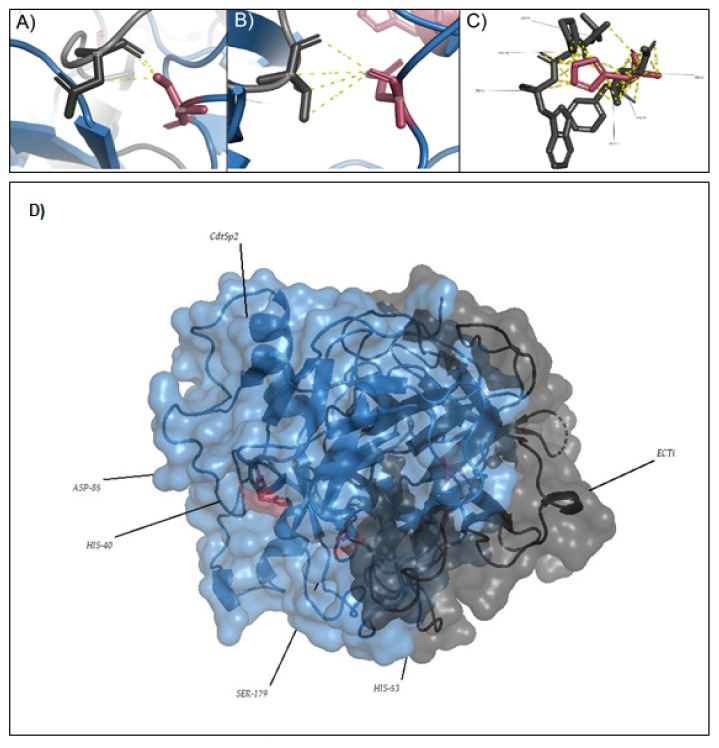
Interaction between EcTI with the amino acid residues of the Active Site of Cdtsp-2. The bonds are 0.9 Å to 3.5 Å. (**A**): His 188. (**B**): Asp 92. (**C**): His 43. (**D**): Interaction between EcTI and Cdtsp-2.

In the interaction of Cdtsp-2 with the EcTI inhibitor simulation (Figure 10D) we found a completely different interaction from the one shown by Zhou [17]. The interaction of Cdtsp-2 with EcTI shows bonds of all types, van der Walls, hydrogen bridges and even covalent bonds in the active site, as we show in Figure 10, whose distances were from 0.9 Å to 3.5 Å. However, the interactions between the protein and the inhibitor occurred through all the amino acids of both proteins, not only between the active site and the reactive loop, as shown by Zhou [17]. It is possible to see all the interactions and alignments that occurred between the amino acid residues of the proteins.

## 3. Discussion

Cdtsp-2 is a protein that closely resembles human Gyroxin and thrombin, and its active sites are highly preserved [6] (Figure 1). The protein can act in the coagulation cascade and in the breakdown of fibrinogen into fibrin monomers. However, Cdtsp-2 is not as glycosylated as Gyroxin. Therefore, it is plausible that protein glycosylations are crucial for its function and to inhibit the action of other elements. Another significant difference between Thrombin and Cdtsp-2 is the type of clot formation; the former produces firm clots, while the latter produces only loose clots.

Batista [15], when isolating EcTI, found that it was possible to inhibit factor XIIa of the coagulation cascade, plasma kallikrein and plasmin, chymotrypsin and trypsin, but it was not possible to inhibit factor Xa and thrombin. However, this result is not seen when compared to Cdtsp-2. Although the active sites are highly preserved in the thrombin mimics, structural changes in the allosteric regions may account for the differences in inhibition of enzyme activity [22,23]. More tests were done to evaluate the pharmacological characteristics of the Cdtsp-2 protein and its possible actions in in vivo tests. As Cdtsp-2 is a recently discovered serine protease [12], not much is known about its activities. In particular, it is not known whether it is hepatotoxic or not. For the in vivo studies, 25 g to 35 g female Swiss mice were used. The paw model described by DI ROSA [24] was used, injecting 10μg of Cdtsp-2 protein into the right paw, and saline (0.9% NaCl) into the intraperitoneal region 10 min later, to mimic a parenteral treatment. The edema test aims to observe the effectiveness of protective treatments, such as the injection of saline, the pure compounds and the inhibitor, 30 min before exposure to Cdtsp-2.

Our group’s study [12] paw edema assay revealed that the serine protease Cdtsp-2 was not only able to induce damage to the coagulation cascade as a thrombin similar, but was also able to produce significant edema, i.e., the protein in question also triggers the inflammatory process. This result, shown in Figure 3, is consistent with Menaldo et al. [25], who showed a serine protease purified from the venom of a snake of the genus Bothrops, which can also cause edema.

The EcTI inhibitor showed a significant ability in reducing edema (Figure 3A). This Kunitz-like inhibitor also acts by decreasing key inflammatory cytokines, such as TGF-α, IL-6, IL-8 and MCP-1, in addition to NFκB, a transcription factor, responsible for aggressive and metastatic characteristics [16]. Cdtsp-2, indeed, presents the ability to cause myonecrosis, as published by Costa and collaborators in 2018; we can observe this in Figure 5. Its ability to cause edema and cell death is quite particular when compared to the other enzymes of the same type, but very similar to the effects caused by sPLA2, already described in the literature [26], and we can see that a inhibitor of sPLA2 the DXPshowed a significant ability in reducing edema from Cdtsp-2 (Figure 4). Besides, CK results showed that there was an expressive reduction in the myotoxicity caused by Cdtsp-2 when exposed to EcTI (Figure 5). The induction of myonecrosis by Cdtsp-2 was very similar to the myonecroses presented by other classes of proteins. 

Accidents with *Crotalus durissus terrificus* can be fatal, since the venom has neurotoxic, myotoxic, coagulant and hepatotoxic actions [9,11]. Therefore, tests with Cdtsp-2 related to hepatotoxicity are necessary, and the inhibitory capacity of EcTI against these parameters is essential for the development of a possible adjuvant treatment. JR Mitchell and co-workers [22] observed in their studies that glutathione depletion by acetaminophen was increased by treatments that potentiate liver necrosis and covalent binding produced by the toxic metabolite of acetaminophen. Due to this fact, many studies now use paracetamol (acetaminophen) as a gold standard for hepatotoxicity. In our work, we also used it as a positive standard.

Lactate dehydrogenase (LDH) is an enzyme present in almost all tissues [27]. Increases can be caused by diseases such as hepatitis, anemia, heart attack, fractures, muscle trauma, cancer and infections such as encephalitis, meningitis and HIV [28]. Since LDH is nonspecific and routine isoenzyme analysis is usually not available in clinical laboratories, LDH measurements provide incomplete data and other tests, such as CK for muscle, ALT for liver, troponin for heart disease, etc. are required. Also, LDH activity is impaired when there is hemolysis in the blood sample. Since red blood cells (RBCs) have the LDH protein, hemolysis leads to false-positive results [29].

Our samples had an elevated concentration of LDH, which despite being a nonspecific test, corroborates with the results we found for CK and all the other hepatitis and inflammatory markers. These data are in line with the results found in the studies of Barravieira and collaborators in [9], France [11] and Al-Quraishy [30]. Still regarding the LDH results, the EcTI inhibitor was unable to reverse the damage already caused by Cdtsp-2 (Figure 5A). Another non-specific marker for hepatotoxicity used was Gamma GT (Gamma-Glutamyltransferase); this marker can be produced in organs such as liver, kidneys, seminal vesicles, spleen, pancreas, heart and brain [31]. It may be increased in renal failure, pancreatic disease, myocardial infarction, diabetes and other diseases besides liver disease.

GGT levels increase markedly in the setting of bile duct obstruction, and the ratio of alanine aminotransferase (ALT) to GGT can guide the clinician in deciding between obstructive jaundice (low ALT/medium-high GGT) versus hepatitis (high ALT/low-medium GGT) during the investigation of a patient with jaundice [32]. Alanine aminotransferase is the most widely used biological indicator for liver health [33]. ALT, as the name suggests, is linked to the transamination of alanine and is concentrated in greater amounts in the liver than in other organs. The release of ALT by the hepatocyte into the bloodstream occurs after hepatocellular injury, and it is eliminated with a plasma half-life of approximately 42 h in humans and 24 h in mice [34]. Elevation of ALT can indicate liver injury, but is not liver-specific. ALT can be caused by muscle, skeletal or cardiac damage and drugs that increase ALT gene expression [35]. 

Aspartate aminotransferase is a transaminase enzyme which catalyzes the reaction between aspartate and alpha-ketoglutarate to form oxaloacetate and glutamate. The enzyme AST, also called serum glutamate oxalate transaminase (SGOT), is present in all tissues except bone and levels are highest in the liver and skeletal muscles. The concentration of AST is increased after injury, trauma, necrosis, infection or neoplasia in the liver or muscle [36]. Another liver marker, C-reactive protein (CRP), was discovered by researchers Tillett and Francis [37] when they identified a substance in the serum of patients with acute inflammation. Our results suggest that there was no bile duct obstruction, since in the results found in Figure 6 the GGT is low-medium, and both aspartate aminotransferase (AST) and alanine aminotransferase (ALT) in Figure 6 are at high levels, thus suggesting hepatotoxicity.

CRP is a protein composed of five subunits synthesized by the liver which reacts to the acute phase of an inflammatory/infection process, mainly due to the action of IL-6 on the gene that controls CRP transcription [38]. In Figure 6C, we show that Cdtsp-2 generated a significant increase in CRP, as much as paracetamol when compared to the untreated animals. Another interesting result shown in the graph was that the Kunitz-like inhibitor EcTI was able to reduce the damage caused by the serine protease (Cdtsp-2); these results corroborate with the studies of T Shimomura and co-workers [39].

Barravieira et al. [7,9] showed in his works that the liver presents alterations after 6 h of snake-venom exposure. However, our samples were exposed to Cdtsp-2 for 4 h, and because of this we cannot see any alterations in histopatology analysis.

Shimomura, in his studies, revealed that the hepatocyte growth factor (HGF) activator is a serine protease produced by the liver that circulates inactive in the blood. 

Shearer and colleagues [40] described in their work that by suppressing PAR2 there was an effective inhibition to in induced fibrosis, inflammation, steatosis and hepatocellular necrosis, thus suggesting a new multifaceted approach to treat severe liver diseases.

**Scheme 1:**
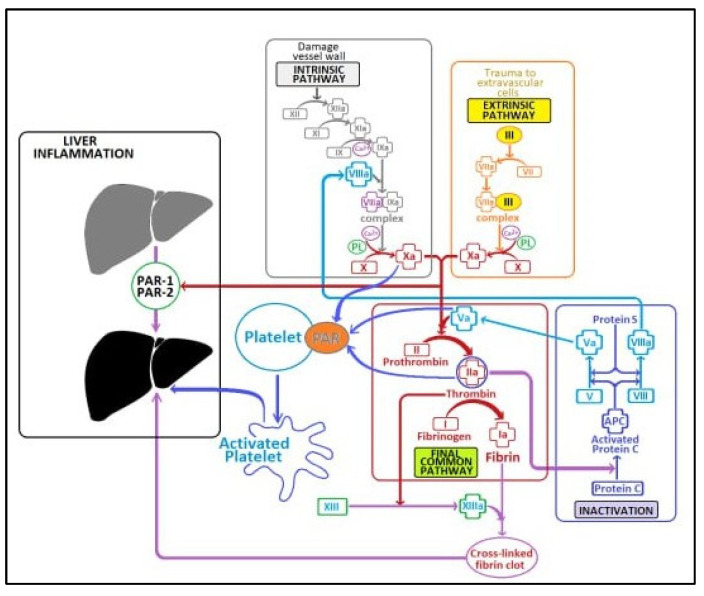
In summary, this scheme shows that Cdtsp-2 is involved with perturbation of coagulation cascade, in activation of platelet and liver inflammation through activation of PAR’s receptors.

Cdtsp-2 is a serine protease that depends on PAR1 and PAR2 to trigger its pharmacological effects [12], thus, our results demonstrate that the EcTI inhibitor was able to strongly interact with Cdtsp-2, not allowing the protease to activate PAR 2 in the liver, thus preventing the hepatobiliary inflammatory cascade (Figure 6, Figure 7 and Figure 8 and Scheme 1).

Tissue injury activates HGF, which is converted to the active form by proteolysis. The activator of HGF activates an inactive protein which is transformed into a biologically active heterodimer in the injured tissue. Activated HGF may be involved in the regeneration of injured tissue. The inhibitor (HGF) is a member of the Kunitz family of serine protease inhibitors, as is EcTI [41].

It is crucial to understand the secondary and tertiary structures of a protein to fully understand its physiological functions. Thus, the circular dichroism assay was performed with the Cdtsp-2 fraction. This experiment shows us that 17.84% of the structure is composed of alpha-helices; 21.16% is in beta sheets and the other amino acids are in random structures (Randon coil), about 61% of the structure (Figure 8A,B). The results that we found through circular dichroism correlate with the structures found in studies conducted by [7,9,22]. The inhibition found by Rodrigues C.F. et al. [23] was associated with the structural changes of Thrombin and the modification in its activity, which occurred by changes in its catalytic site or allosteric regions, which did not occur with Cdtsp-2, possibly due to the absence of glycosylations.

The model of Cdtsp-2 is consistent with sequencing and circular dichroism, which shows a predominance of the secondary structure random coil, different from the serinoprotease already described for *Crotalus durissus terrificus* venom. The percentage of serine proteases in the venom increased from 2.5% to about 7% of the total [12], increasing the problematic nature of the antivenom serum, since serine proteases are not fully neutralized in their enzymatic and pharmacological activity by the antivenom serum [42].

Typically, inhibition of trypsin-like serine proteases is through the interaction of the reactive loop with the active site cleft [43]. The Kunitz-like inhibitor (EcTI), when exposed to serine protease trypsin, forms a complex with the standard inhibitory mechanism in which the reactive loop of the inhibitor is anchored to the active site of the trypsin, with Arg64 and Ile65 side chains occupying S1 and S1’ pockets, respectively [17]. However, when we study interactions with thrombin simile-type proteins, the forms of interaction and inhibition are different.

Otlewski and co-workers [44] also summarized the types of interactions that occur between inhibitors and thrombin simile-type serine proteases. One relevant example is direct active site blockade. This type of inhibition is given by non-canonical inhibitors of serine proteases. These inhibitors insert their N-terminal tail into the active site of the enzyme, forming a small β-sheet parallel with the enzyme residues. Non-canonical inhibitors were created in hematophagous animals as anticoagulants to inhibit thrombin or factor X a. The paradigmatic example is the recognition of thrombin by the leech hirudin inhibitor. The N-terminal end of the globular domain of hirudin establishes contact with the active site through the parallel β-sheet, while the C-terminal acidic end is recognized by the anionic fibrinogen recognition exosite [44]. 

According to Richardson and co-workers (2000), the hemedin of a terrestrial leech makes interaction through the N-terminal in a similar way to that cited by Gutter [44], but the acidic C-terminal segment interacts with the heparin binding surface, which differs from the two interaction models [45]. In both examples, the inhibitors form a monomer-like protein complex, as found in the protein-protein docking shown in Figure 8D, i.e., Cdtsp-2 interacted with the EcTI inhibitor like thrombin interacts with inhibitors from blood-phage animals; this result is compatible with those found in the strong enzymatic and pharmacological inhibitions against Cdtsp-2.

## 4. Materials and Methods

### 4.1. Purification of Cdtsp-2 

The purification of the Serine Portease Cdtsp-2 was done following the methodology of Fonseca et al. [46] and Costa et al. [12].

### 4.2. Reagentes 

We obtained commercially from Sigma-Aldrich (St. Louis, MO, USA) all other chemicals, reagents and kits were purchased from Sigma-Aldrich^®^, Bio-Rad (Hercules, CA, USA), Cayman Chemical (Ann Arbor, MI, USA).

### 4.3. Enzyme Assay of Cdtsp-2 Inhibition

The inhibition potential of the EcTI inhibitor was checked against Cdtsp-2. The enzyme was dissolved in 0.9% NaCl saline solution, with a final concentration of 1 mg/mL. The samples were incubated with the pure compounds for 20 min at room temperature, then the solution was incubated on a microplate together with the substrate BapNa, solubilized in water at 37 °C and buffer 50 mM Tris-HCl, pH 7.4, 100 mM NaCl, plus a control (blank − substrate + saline + buffer) and positive control. The samples were read at sequential intervals (5 min, with a total time of 90 min) in a spectrophotometer for reading (λ = 405 nm) (adapted from Prasa et al. [18]) and all absorbance data were transformed in velocity using this formula: V_0_ = nm.258 mmol/Tempo(min).

### 4.4. Circular Dichroism

Polarized light is used in the distal ultraviolet (UV) range (from 180–260 nm). This technique allows the evaluation of the structural integrity of proteins, conformational changes, processes of denaturation (unfolding) and renaturation (folding), making it possible to estimate the composition of the elements in the secondary structure of this macromolecule. The circular dichroism analysis was performed with 0.02 mg/mL of Cdtsp-2 monitored using wavelengths of 190–260 nm. The analysis was performed to 8 convolutions, and data were treated by Spectra manager.

### 4.5. Evaluation of Paw Edema

In vivo experimental models were performed to evaluate inhibition of acute inflammation caused by purified Cdtsp-2 using randomly chosen Swiss mice (~25 g, *n* = 5). These assays were performed only after in vitro investigation of inhibition or interaction of the compounds with the enzymes used in this work. Animals were treated with 50 μL (0.5 μg/μg per animal/10 μg per animal) of the compounds via peritoneal or intravenous injections. A saline solution (0.9% NaCl) was used for the controls. There were 5 groups: Saline, Cdtsp-2, Cdtsp-2 previously incubated with EcTI, EcTI and carrageenin (1.5% in solution, with positive control). Edema volume monitoring was achieved using a digital plethysmometer for about four hours. After the tests, the mice were anaesthetized and sacrificed via cervical dislocation. In vivo experiments were performed according to the institutional rules and were approved by the ethics committee from UNESP, number 10/2018-CEUA.

### 4.6. Hepatotoxicity

The hepatotoxicity study was performed using the methodology according to Hewedy (2021) [47], where hepatic injury was induced by intraperionial injection using as positive gold standard the acetaminophen molecule (paracetamol, N-acetyl-p-aminophenol). Fifty µg/µL of the EcTI inhibitor, previously incubated for 30 min with Cdtsp-2 (1:1 *w*/*w*), were injected into the right peritoneum (*n* = 5). After 30 min on average, blood from the animals was collected from the tail in a heparinized tube, which was centrifuged and frozen for further testing. Control animals (*n* = 5) were subjected to the same procedure as treated animals, but were only inoculated with saline, pure compounds and inhibitor. The observation time of the animals was 4 h. After testing, the mice were anesthetized and sacrificed via cervical dislocation. With the separated serum we used commercial kits where we followed the manufacturers’ guidelines for the determination of CRP (C-reactive protein), AST (aspartate aminotransferase), ALT (alanine aminotransferase, LDH (lactate dehydrogenase) and gamma-GT (gamma glutamyl transferase).

#### Histopathology

Livers were collected and their wet weights (absolute and relative to body weight) were recorded and destined for histological procedures. Samples were fixed in buffered formaldehyde solution (10%) for 24 h and lately transferred to 70% alcohol. The pieces were processed according to the method described by Medeiros et al. [19] and histopathological evaluations of the livers were made qualitatively with the aid of a light microscope in a blinded trial, based on the guidelines issued by the Society for Toxicologic Pathology [20] and the National Toxicology Program Health and Human Services [21]. The following parameters were observed for hepatic tissue: presence of hydropic or fat degeneration, defense cells foci, mitotic figures and binucleated hepatocytes and hyaline casts. All parameters were classified according to the frequency: absent, mild (<25% of the histological section), moderate (25% to 50% of the histological section) and severe (occurring between 50% and 100% of the histological section).

### 4.7. CK Level Measurement

In total, 20 μL (2.5 μg) of the compounds were added into the peritoneum 10 min after Cdtsp-2 (*n* = 5) was injected into the gastrocnemius muscle. After an average of 30 min, blood from the animals was collected from the tail in a heparinized tube, which was centrifuged and frozen for later analysis. Control groups (*n* = 5) underwent the same procedure as the treated animals, but were inoculated with 20 μL of 0.9% NaCl, 20 μL (2.5 μg) of the purified compounds or 20 μL (10 μg) of isolated Cdtsp-2. After testing, the mice were sacrificed by cervical dislocation. Serum creatine kinase (CK) levels were determined according to the kit manufacturer (Bioliquid, Pinhais, Brazil).

### 4.8. Statistical Analysis

Data are expressed as mean ± standard deviations. The results were analysed through an analysis of variance (ANOVA) of one or two routes followed by the Bonferroni and t-test with statistical variance *p* < 0.05.

### 4.9. Preparation of Ligands

Thrombin (1doj), Trypsin (6yiw), Cdtsp-2 (Gka1) and EcTI (4j2k) proteins were found in PDB (Protein Data Bank-https://www.rcsb.org accessed on 18 September 2022) and NCBI (NCBI https://www.ncbi.nlm.nih.gov accessed on 18 September 2022) database and coding was used in this analysis to find the amino acid sequence of all the proteins. The crystallographic model was chosen as the best model for building the theoretical structural model from human models.

The structure information of the compounds was taken from the PubChem platform (https://pubchem.ncbi.nlm.nih.gov accessed on 20 September 2022) associated with the Molinspiration and SwissADME platforms were used for better visualization of the structure of the compounds [40]. The ligands were in 3D format with the SDF extension. After downloading, the ligands and files were converted to Mol2. format and the PDB models were generated by RCBS, to be inserted into the SwissDock platform for further analysis in the Chimera 1.14 tool, where we observed the sites that have the highest interactions between the compound and the protein, among the 250 positions generated by SwissDock in this preliminary docking [42]. 

The Swiss ADME platform (www.swissadme.ch accessed on 20 January 2023) was created by the Swiss bioinformatics institute. The platform was used to estimate individual types of ADME biological behavior, prior to chemical synthesis and biological testing, to approximate the biological activity profiles of the simulated molecules [46].

### 4.10. Molecular Docking

In this study, the molecular docking experiments were performed by the SwissDock platform [48], subsequently we used the CavityPlus platform [49], following the idea of XU et al. [50] that the association of using the platforms generated better results to generate a Grid Box more faithful to reality, using the Autodock Vina software [49,50]. Several results from various fitting runs were generated and summarized in a table for further analysis [45,47].

The SwissDock and Cavity Plys platform (https://swissmodel.expasy.org, accessed on 16 April 2023) was used to assemble the structural molecular model of the protein and evaluate the possibilities of binding the chosen protein.

After a prior study of the mechanisms and essential residues of these proteins, molecular docking experiments were performed by Autodock Vina with two Grid Boxes: one larger, covering more site residues, and the other with the 40 × 40 × 40 parameters focused on the site of largest interactions with 100 exhaustive assays per sample [51].

Coupling calculations using the Lamarckian genetic algorithm and standard procedures for fitting a versatile ligand to a rigid protein were performed with AutoDock 5.6. The docking calculations were performed on the binding of each protein target and catalytic site.

Once the possible binding sites were identified, the coupling of compounds to these sites was performed to decide the most likely and most energetically desirable binding conformations [43].

Autodock Vina 1.1.2 was used to obtain robust docking simulations involving a grid box at the identified binding site. A 32 Å matrix with a grid spacing of 0.375 Å was bounded by an active site. Affinity scores were obtained and ranked based on the free energy binding theory provided by AutoDock Vina (in kcal/mol). To verify the binding relationships, we used Discovery Studio Visualizer 2.5 (http://3dsbiovia.com/products/ accessed on 16 April 2023), PyMOL and LigPlot+ [52,53], evaluating the binding energies, distances and orientations of the molecules in the microenvironment of the active site of the enzymes with the binding compounds [50].

## 5. Conclusions

The Kunitz-like inhibitor purified from *Enterolobium contortisiliquum* EcTI was efficient against the damage caused by serine protease. These results were important pointers for studies on the mechanism of interaction between Cdtsp-2 and EcTI proteins. The EcTI inhibitor showed strong interactions not only with the triad of the catalytic site, but also with all other amino acids of the protein, forming a complex and inactivating the enzymatic activities of Cdtsp-2. Therefore, we can see that Kunitz inhibitor is a plausible option for developing ancillary treatments against the biological activities of venoms.

## Data Availability

Data are in the article.
